# Effect of siRNA against NF-κB on sepsis-induced acute lung injury in a mouse model

**DOI:** 10.3892/mmr.2014.2299

**Published:** 2014-06-05

**Authors:** LI-YAN JIN, CONG-FENG LI, GUANG-FA ZHU, CHUN-TING WU, JUN WANG, SHU-FENG YAN

**Affiliations:** 1 Department of Infectious Disease, Beijing Anzhen Hospital, Capital Medical University, Beijing Institute of Heart, Lung and Blood Vessel Diseases, Beijing 100029, P.R. China; 2 Department of Respiratory and Critical Care Medicine, Beijing Anzhen Hospital, Capital Medical University, Beijing Institute of Heart, Lung and Blood Vessel Diseases, Beijing 100029, P.R. China; 3 Department of Respiratory and Critical Care Medicine, Beijing Luohe Hospital, Capital Medical University, Beijing 101100, P.R. China; 4 Department of Critical Care Medicine, Beijing Jishuitan Hospital, 4th Clinical Medical College of Peking University, Beijing 100035, P.R. China

**Keywords:** small interfering RNA, NF-κB, sepsis-induced acute lung injury

## Abstract

The aim of the present study was to explore the protective effect of small interfering RNA (siRNA) against nuclear factor κB (NF-κB) p65 on sepsis-induced acute lung injury (ALI) in mice. In total, 70 male Kunming mice were randomly divided into a healthy control group, a sepsis group, a specific interfering group and a scrambled control group (Sc), and the latter three groups were divided into post-operational 6 and 12 h subgroups, each of which consisted of 10 mice. The mice were administered with NF-κB siRNA, scrambled siRNA and normal saline via tail vein injection. Following 1 h, a mouse model of septic ALI was produced by cecal ligation and puncture (CLP) in the two siRNA groups and the sepsis control group. At 6 and 12 h post-operation, the experimental mice were sacrificed and the lung tissue samples were collected. Histopathological changes, wet/dry ratio of lung weight, NF-κB protein and NF-κB p65 mRNA levels, matrix metalloproteinase-9 (MMP-9) mRNA and protein activity were detected. Compared with the sepsis group and the Sc at the corresponding time, the expression levels of NF-κB p65 mRNA, the lung injury of experimental mice, the wet/dry ratio and the levels of MMP-9 mRNA and protein activity decreased, and significant differences were observed at 6 h post-operation (P<0.05). RNA interference against NF-κB p65 was able to decrease the expression of NF-κB and further inhibit the early phasic excessive inflammatory reaction in sepsis, which may alleviate ALI.

## Introduction

Nuclear factor κB (NF-κB) is an ubiquitous transcription factor, which is involved in the regulation of gene expression in cytokines and pathways associated with inflammation, and can delay the apoptosis of neutrophils (1,2). The regulation or inhibition of the excessive activation of NF-κB are effective methods to control diseases associated with inflammation, including acute lung injury (ALI). ALI is a clinical syndrome associated with respiratory dysfunction and is usually a consequence of sepsis and a systemic inflammatory response. The most severe form of ALI has a mortality rate of 30–50% and, with the exception of low volume ventilation, no interventions have been demonstrated to be effective (3,4). Since the most common cause of ALI in humans is sepsis, infusion of gram-negative bacterial endotoxin has been used as a model of sepsis-associated lung injury.

During sepsis, pro-inflammatory cytokines, including tumor necrosis factor (TNF)-α, interleukin (IL)-6 and IL-17, are markedly upregulated. These mediators are known to contribute to the vascular abnormalities and changes in bronchomotor tone which occur in ALI. Previous results using other types of macrophages have demonstrated that the expression of the pro-inflammatory cytokines were regulated by the activation of NF-κB (5,6).

RNA interference (RNAi) can specifically inhibit the transcription of target genes, thus reducing the corresponding protein levels, and has a high efficiency and specificity (7,8). In the present study, cecal ligation and puncture (CLP) were used to stimulate sepsis in order to establish a model of ALI. In addition, NF-κB p65 small interfering RNA (siRNA) was transfected into mice, and the effect on lung injury as well as the expression of NF-κB, matrix metalloproteinase-9 (MMP-9) and interleukins were examined.

## Materials and methods

### Materials

The pSUPER.retro.neo (VEC-PRT-0003 linear) plasmid DNA and the retrovirus packaging cell line 293E were kindly provided by Dr Yang Yizeng (NIH Center for Molecular Studies in Digestive and Liver Diseases, University of Pennsylvania, Perelman School of Medicine, Philadelphia, PA, USA). The mouse monocyte-macrophage J774A.1 cells were purchased from the Institute of Basic Medical Sciences, Peking Union Medical College (Beijing, China).

Male Kunming mice, weighing 18–20g, were purchased from the Experimental Animal Center of the Chinese People’s Liberation Army Academy of Military Medical Sciences (Beijing, China). The pathogen-free animals were maintained at 22°C on a 12-hr light/dark cycle with free access to water and a standard rodent diet. All the procedures with animals were performed in accordance with the Harvard Medical School Office for Research Subject Protection (Boston, MA, USA) and were approved by the Ethics Committee of the Chinese National Genome Center (Shanghai, China). Fetal bovine serum was purchased from Gibco-BRL (Carlsbad, CA, USA). DMEM, Opti-MEM medium and trypsin were purchased from HyClone (Logan, UT, USA). Lipofectamine 2000™ was purchased from Invitrogen Life Technologies (Carlsbad, CA, USA). Plasmid extraction kits (no. 845-KS-5040050 innuPREP Plasmid Mini kit) were obtained from Shengong (Shanghai, China). All restriction endonucleases and T4 DNA ligases used were purchased from MBI Products (St, Elyria, OH, USA). The AMV reverse transcriptase kit was purchased from Promega Corporation (Madison, WI, USA). NF-κB p65 and mouse anti-human polyclonal antibody were purchased from Santa Cruz Biotechnology, Inc. (Santa Cruz, CA, USA). Goat anti-mouse immunoglobulin (IG) G antibody was obtained from the Zhongshan Haiji Medical Company Biology Engineering Co., Ltd. (Beijing, China). The ELISA kit was purchased from Huayue (Guangzhou, China). Coomassie Brilliant Blue R-250 was purchased from Amresco (Solon, OH, USA). The surgical instruments were purchased from Shanghai Medical Instruments Co., Ltd. (Shanghai, China).

### Methods

#### Plasmid construction

NF-κB p65 siRNA retroviral vectors were constructed according to the sequence information from GenBank (gene no. M61909). RNAi design software (Invitrogen Life Technologies) was applied for the sequence design. Based on the vector sequence, a NF-κB p65 siRNA target sequence and a randomly distributed control sequence were designed.

The pSUPER.retro.neo vector was double digested with *Bgl*II and *Hind*III. The siRNA target sequence and the randomly distributed sequence were connected to the linearized plasmid vector and transformed into competent bacteria *E. coli* DH5α. Single colonies were picked, the plasmid was extracted and digested, and then electrophoresis and gene sequencing was performed.

### Cell culture

293E cell culture and transfection: 293E cells were recovered and cultured in the DMEM medium with 10% fetal bovine serum in 5% CO_2_ in a 37°C cell incubator. 293E cells were passaged every three days. Prior to transfection, cells were washed three times with sterile PBS or replaced with serum-free DMEM medium and cultured for 6 to 8 h in order to remove the effect of the serum protein. The transfection procedure was as follows: i) Plasmids (24 μg), including the siRNA sequence or the scrambled sequence were diluted with 1.5 ml of Opti-MEM and mixed gently; ii) Lipofectamine™ 2000 (60 μl) was diluted with 1.5 ml of Opti-MEM, mixed gently and incubated for 5 min at room temperature; iii) the premixing products were mixed and incubated for 15–25 min at room temperature; iv) DNA-Lipofectamine™ 2000 (3 ml) was added to each plate and then an additional 3–5 ml of Opti-MEM was added and the plate was agitated gently to promote mixing; v) the medium was replaced with serum-free DMEM medium for 6–8 h and cultured in a CO_2_ incubator at 37°C, and the virus supernatant was collected following 24, 48 or 72 h and named the siRNA group and the scrambled group. Virus titration was performed with NIH3T3 cells. The siRNA viral titer was 3.94×10^6^ colony forming units (CFU)/ml and the scramble viral titer was 3.62×10^6^ CFU/ml.

### Animal experiment

The sepsis was induced by cecal ligation and puncture as previously described (9). Briefly, animals were weighed and anesthetized using sodium pentobarbital (intraperitoneally, 40 mg/kg). The lower abdomen area was shaved and disinfected; a median 0.5–1.0 cm incision was made in the lower abdomen. After careful dissection, the cecum was ligated below the ileocecal valve, followed by a single ‘through and through’ perforation (21-gauge needle); 75% of the cecum was ligated to establish the sepsis model and the control group received no cecal ligation and puncture. Normal saline was administrated subcutaneously (50 mg/kg) to replenish bodily fluid after the surgery. Then the mice were treated with different viruses containing NF-κB p65 siRNA or control scramble RNA via tail vein injection. Samples were collected from each group of mice for assessment of the lung injury at different time points.

In order to assess the severity of the lung injury, a semi-quantitative histological index of quantitative assessment (IQA) of lung injury was used. Eight sections were randomly selected from each group of rats, and 10 fields from each section were examined by microscopy (x40). A pathologist who was blinded to this study evaluated all of the sections. The average values of the lung injury obtained were considered a semi-quantitative histological IQA of lung injury.

### Quantitative polymerase chain reaction (qPCR) analysis

Total RNA was extracted from pulmonary tissues using TRIzol reagent (Invitrogen Life Technologies) according to the manufacturer’s instructions. qPCR kits (Takara, Kyoto, Japan) were used for the qPCR experiment. Total RNA (4 μg) templates were used to make cDNA by using AMV reverse transcriptase and random primers (9-mer) as the first strand primer. Synthesized cDNA was used in qPCR experiments. qPCR was performed using a 40-cycle two-step PCR with sequence-specific primer pairs using the ABI 7900 fast real-time detection system (Invitrogen Life Technologies). The qPCR cycling conditions were: 95°C for 10 min, 40 cycles of 95°C for 15 sec and 60°C for 1 min, then 95°C for 1 min, followed by dissociation curve analysis. The qPCR reagents used in were from the GoTaq^®^ qPCR Master Mix (A6001; Promega Corporta). Primers were designed using the Primer Express 3.0 software and the sequences were as follows: MMP9: Forward, 5′-AAAGACCTGAAAACCTCCAACCT-3′, and reverse, 5′-GCCCGGGTGTAACCATAGC-3′; TNF-α: forward, 5′-GAAGTTCCCAAATGGCCTCC-3′, and reverse, 5′-GTGAGGGTCTGGGCCATAGA-3′; P65: forward, 5′-GGTCCACGGCGGACCGGT-3′, and reverse, 5′-GACCCCGAGAACGTGGTGCGC-3′. The levels of mRNA expression were evaluated as a ratio based on the qPCR results for lung tissue GAPDH mRNA using the 2^−ΔΔCt^ method.

### Western blot analysis

The amount of NF-κB protein was detected in J774A.1 cells using western blot analysis. The two groups of cells were stimulated with 50 ng/ml of lipopolysaccharide (LPS) and the total protein was extracted 2, 6, 12, 24 h after stimulation with radioimmunoprecipitation assay (RIPA) lysis buffer. Then the samples were detected using western blot analysis. Total protein (50 μg) was loaded in each lane. The proteins were transferred onto polyvinylidene fluoride membranes (PVDF) using a wet transfer method following polyacrylamide gel electrophoresis, then blocked with 3% bovine serum albumin for 1 h at room temperature and washed with TBST three times. The PVDF membrane was incubated with α-NF-κB p65 antibody (1:500) overnight at 4°C. The membrane was washed with TBST and incubated with secondary antibody (goat anti-mouse IgG 1:5,000) for 2 h at 4°C. Then the membrane was washed with TBST three times for developing, fixing and exposure. The experiment was repeated three times.

### ELISA

Levels TNF-α, IL-6 and IL-17 was detected using ELISA according to the manufacturer’s instructions. The experiment was repeated three times and results are presented as the mean value.

### Histological examination

The right lower lung was inflated and fixed with 4% buffer formalin for 24 h. Routine paraffin-embedded tissue sections (5 μm) were prepared and stained with hematoxylin and eosin (H&E) for examination under an optical microscope (BX51; Olympus, Tokyo, Japan).

### Lung wet/dry weight ratio

The superior lobe of the right lung was cleansed and weighed to obtain the wet weight and was then placed in an oven at 80°C for 48 h for the measurement of the dry weight. The ratio of the wet weight to dry weight was calculated to assess the tissue edema.

### Detection of matrix metalloproteinase-9 (MMP-9) activity in lung tissue using gelatin zymography

The lung tissues (20 mg) were collected 6 and 12 h after surgery, the total protein was purified with RIPA lysis buffer and the proteins were separated by SDS-PAGE. The gel was washed with 2.5% Triton X-100 solution and incubated at 37°C for 18 h following washing. The gel was stained with Coomassie Brilliant Blue R-250 staining solution for 2 h and then destained with 30, 20 and 10% destaining solution for 30 min for 1 and 2 h, respectively. The transparent clear target bands in the blue background were observed. Finally, the gray value was analyzed with image processing software (ImageJ 1.48; National Institutes of Health, Bethesda, MD, USA).

### Statistical analysis

For comparisons between groups, paired Mann-Whitney U tests or unpaired Students’ t-tests with or without Welch correction and one-way analysis of variance were used. P<0.05 was considered to indicate a statistically significant difference. The statistics were calculated using GraphPad Prism (GraphPad Software, Inc., La Jolla, CA, USA).

## Results

### Expression of NF-κB and TNF-α was inhibited by the NF-κB p65 siRNA retrovirus in vitro

**Figure 1:**
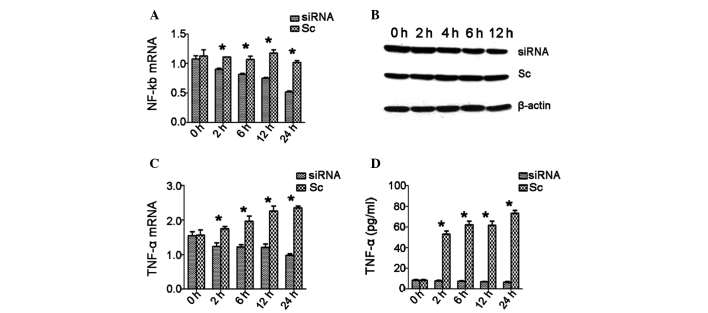
Time-dependent effect of NF-κB siRNA on J774A.1 cells *in vitro*. Levels of (A) NF-κB mRNA and (B) NF-κB protein at different time-points. Levels of (C) TNF-α mRNA (D) and TNF-α protein following stimulation with lipopolysaccharide. Data are expressed as the mean + standard error (n=6). ^*^P<0.05 compared with Sc. NF-κB, nuclear factor κB; TNF-α, tumor necrosis factor-α; siRNA, small interfering RNA; Sc, scrambled control group.

NF-κB is an important transcription factor, which is involved in the regulation of gene expression in cytokines and inflammation-associated pathways. In order to inhibit the expression of NF-κB, NF-κB p65 siRNA retroviruses were constructed. It was revealed that the expression of NF-κB mRNA and protein was decreased in J774A.1 cells 2 h after viral infection (Fig. 1A and B). In addition, the expression levels decreased gradually as the incubation period prolonged. Furthermore, it was revealed that the expression and secretion of TNF-α in siRNA-transfected cells decreased, while TNF-α levels in the Sc cells gradually increased following stimulation with LPS. The ELISA results indicated that the amount of TNF-α siRNA-transfected cells decreased 2 h after LPS treatment (Fig. 1C and D).

### CLP surgery induces ALI and pathological changes in lung tissue

**Figure 2:**
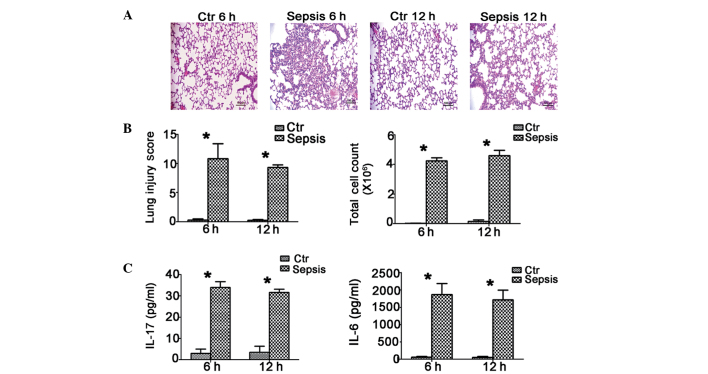
Histological examination of the lung sections at 6 and 12 h after CLP-induced sepsis. (A) Lung tissue from the control and sepsis groups at 6 and 12 h after surgery (hematoxylin & eosin staining; magnification, ×200); (B) Lung injury score and total cell number in the BAL fluid from different groups; (C) Concentration of IL-17 and IL-6 in the BAL fluid from differently treated mice. Data are expressed as the mean + standard error (n=10 per group). ^*^P<0.05 compared with control. IL, interleukin; CLP, cecal ligation and puncture; BAL, bronchoalveolar lavage; Ctr, control.

ALI was successfully induced by CLP surgery. Mice from the sepsis group appeared septic 3 h after surgery. The mice exhibited numerous symptoms, including a curled up back, reduced activity, a reduced response to sound and other stimuli, a reduced appetite, piloerection, shortness of breath, appearance of nasal secretion and eye discharge. The results are shown in Fig. 2. Histological analysis following H&E staining revealed exudative changes, patchy hemorrhage, thickened alveolar interstitium and heavy infiltration of inflammatory neutrophils and lymphocytes into the intra-alveolar and intersititial spaces 6 h following CLP surgery (Fig. 2A). To further assess the degree of pulmonary injury, the lung injury in the lung tissue was scored in each mouse (Fig. 2B). The lung injury scores reached their highest value at 6h after surgery. In addition, the number of total cells, IL-17 and IL-6 in the bronchoalveolar lavage (BAL) fluid were measured. The total cell number in the BAL fluid increased by ~20-fold 6 and 12 h after surgery (Fig. 2B), and this increase was largely due to the infiltration of inflammatory cells. In addition, the levels of IL-17 and IL-6 in the BAL fluid were significantly elevated following CLP surgery (Fig. 2C).

### Effect of NF-κB p65 siRNA on the lung histopathology and lung injury

**Figure 3:**
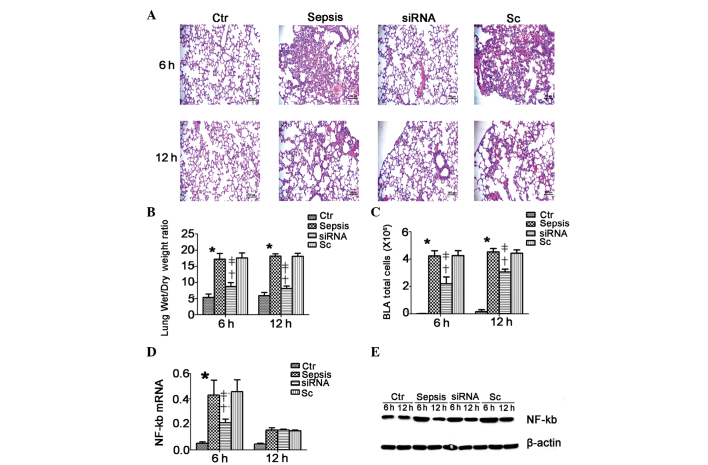
Histological examination of the lung sections at 6 and 12 h after different treatments of mice with CLP-induced ALI. (A) Lung tissue from the control, sepsis, siRNA and Sc groups at 6 and 12 h after surgery (hematoxylin & eosin staining; magnification, ×200); (B) wet/dry ratio of lungs from the control, sepsis, siRNA and Sc groups at 6 and 12 h after surgery; (C) total cell number in the BAL fluid of mice from different groups; (D and E) levels of NF-κB mRNA and protein in the lung tissue of mice from different groups. Data are expressed as the mean + standard error (n=10 per group). ^*^P<0.05 compared with the Ctr group; ^†^P<0.05 compared with the sepsis group; ^‡^P<0.05 compared with the Sc group. siRNA, small interfering RNA; CLP, cecal ligation and puncture; ALI, acute lung injury; Sc, scrambled control group; Ctr, control group; NF-κB, nuclear factor κB; BAL, bronchoalveolar lavage.

The effect of NF-κB siRNA on the lung histopathology is shown in Fig. 3. The treatment with NF-κB siRNA significantly reduced the infiltration of inflammatory cells and improved the histology of the experimental group compared with the siRNA scrambled control (Sc) group at 6h (Fig. 3A). In addition, the administration of NF-κB siRNA markedly reduced the total cell number in the BAL fluid from 4.2 to 2.3×10^6^ 6 h after injection (Fig. 3C). The injection of NF-κB siRNA retroviruses also reduced the lung wet/dry ratio (Fig. 3B) and the difference between the siRNA- and the Sc-treated groups was significant (P<0.05). In order to measure the expression of NF-κB in the lung tissue of different groups, the mRNA and protein levels of NF-κB were detected in the lung tissue of the mice. The expression of NF-κB was upregulated in the lungs of the sepsis group mice compared with those of the control group; however, the administration of NF-κB-specific siRNA retroviruses markedly inhibited the mRNA and protein expression of NF-κB in the lung tissue of mice (Fig. 3D and E).

### MMP-9, IL-17 and IL-6 levels in lung tissue

**Figure 4:**
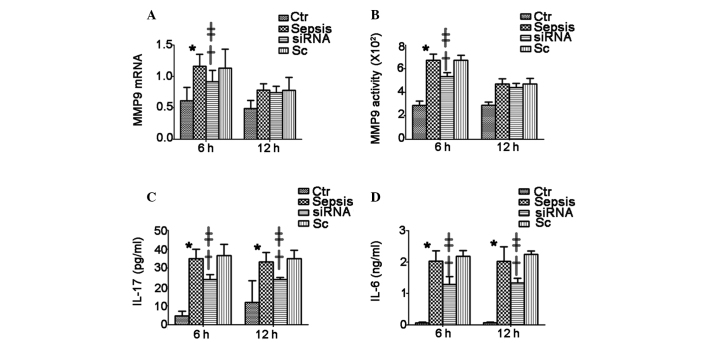
Expression and activity of MMP-9 in the lung and the pro-inflammatory cytokines in the BAL fluid. (A and B) Levels of MMP-9 mRNA and activity in the lung tissue from differently treated mice; (C and D) Concentration of IL-17 and IL-6 in BAL fluid from differently treated mice. Data are expressed as the mean + standard error (n=10 per group). ^*^P<0.05 compared with the Ctr group mice; ^†^P<0.05 compared with the sepsis group mice; ^‡^P<0.05 compared with the Sc group mice. IL, interleukin; MMP-9, matrix metalloproteinase 9; BAL, bronchoalveolar lavage; Ctr, control; Sc, scrambled control group; siRNA, small interfering RNA.

Mice which received the siRNA injection demonstrated lower levels of IL-17 and IL-6 compared with the Sc injection group and the sepsis group at 6 h. However, the levels of IL-17 and IL-6 at 12 h after administration were similar to those at 6h (all P<0.01, 12 h vs. 6 h; Fig. 4C and D).

In addition, the mRNA levels of MMP-9 decreased 6 h after siRNA injection; however, the expression of MMP-9 in the siRNA-treated group demonstrated no change compared with the Sc-treated group at 12 h (Fig. 4A). In addition, the activity of MMP-9 in the lung tissue was decreased in the siRNA-treated group at 6h; however, the MMP-9 activity did not change at 12 h (Fig. 4B). In conclusion, the inhibition of the expression of NF-κB had a suppressive effect on the expression of inflammatory cytokines, including IL-17 and IL-6 and the effect also suppressed the expression and the activity of MMP-9 in lung tissue.

## Discussion

ALI is a condition of acute respiratory failure, characterized by diffuse pulmonary infiltrates and severe hypoxemia. It is usually accompanied by pulmonary edema and the reduction of pulmonary compliance and functional residual capacity (10). A large number of inflammatory cytokines are released into the intracellular and extracellular matrix, leading to lung tissue damage, alveolar membrane damage and alveolar-capillary diffusion dysfunction, causing persistent hypoxemia (11).

Previous reports suggested that monocyte-macrophage system activation and migration leads to the excessive release of inflammatory mediators, including TNF-α, IL-1, IL-6 and IL-8, which are important inducers of diffuse tissue injury and dysfunction. In addition, the excessive release of inflammatory factors is closely associated with the excessive activation of the nuclear transcription factor NF-κB (12–14). The inhibition of NF-κB activity and the reduction of the excessive release of inflammatory factors are important methods for the clinical treatment of ALI.

NF-κB is one of the major transcription factors involved in the regulation of the expression of inflammatory cytokines, a number of pro-inflammatory cytokines, inflammatory mediators, adhesion molecules and acute phase response proteins necessary for the expression of transcription factors. The activated NF-κB can in turn activate and control a variety of inflammatory factors as well as regulate gene expression of a variety of enzymes involved in inflammation amplification and persistence. In previous years, a large number of studies have demonstrated that the NF-κB signal transduction pathway is a hub of a variety of inflammatory signal transduction pathways and the majority of the inflammatory gene activation is dependent on the activation of the NF-κB signaling pathway. NF-κB signal activation and reactivation is important in the early inflammatory response (15).

As an important pro-inflammatory cytokine downstream of the NF-κB pathway, TNF-α is considered to be one of the most important inflammatory factors in the occurrence and persistence of ALI (16). It has been reported that TNF-α can induce the production and release of inflammatory cytokines, including IL-6 and IL-8, in the ALI process. In addition, it can also induce chemotaxis, the activation of neutrophils and the production of oxygen radicals and proteases, which directly damage the cells in tissues. Therefore, the disturbance of this ‘vicious cycle’ in the TNF-α-induced inflammatory response (17) can reduce the inflammation caused by diffuse tissue damage.

MMPs are a class of endopeptidases, which share structural and functional similarities with each other. MMP-9 is a type of matrix metalloproteinase derived from monocytes, macrophages and neutrophils. It can act on gelatin, collagen and proteoglycan. Under physiological conditions, stromal cells can produce low levels of MMPs to constantly promote the removal of foreign substances and hazardous materials. The activity of MMPs is regulated by the tissue inhibitor of metalloproteinases (TIMP) and α2-macroglobulin. A dynamic balance between MMP and TIMP is important for maintaining the structural integrity of the extracellular matrix and the normal function of cells. Previous studies have suggested that MMP-9 is released from alveolar macrophages and neutrophils, which is stimulated by lipopolysaccharide (LPS) and TNF-α (18). The excessive activation of MMP-9 can damage structures, including the basement membrane of lung tissue, thus promoting inflammation (19,20). MMP-9 is closely associated with tissue injury, including enhanced permeability of the alveolar-capillary barrier, exudation of inflammatory cells and factors and destruction of the extracellular matrix following ALI.

In the present study, the NF-κB p65 subunit was selected as a target gene and pSUPER.retro.neo was used as an expression vector. In the J774A.1 cells transfected with the retroviral vector expressing siRNA against murine NF-κB p65, the expression of NF-κB p65 mRNA decreased 2 h after stimulation with LPS and decreased gradually over time. The protein levels of NF-κB p65 in siRNA-transfected cells were also significantly lower than those in cells from the Sc group (Fig. 1A and B; P<0.05) 2, 4, 6 and 12 h after stimulation with LPS, which indicated that the siRNA effectively downregulated the NF-κB levels. The present study also revealed that the expression levels of TNF-α mRNA in siRNA-transfected cells decreased following stimulation with LPS, while the TNF-α mRNA levels increased in the Sc group. Following stimulation with LPS for 2, 4, 6 or 12 h, the expression levels of TNF-α in the siRNA group exhibited statistically significant differences from the Sc group (Fig. 1C and D; P<0.01). ELISA detection of mouse monocyte-macrophage cell supernatants demonstrated that the amount of TNF-α in siRNA–transfected cells decreased 2 h after LPS treatment, while the amount of TNF-α in the Sc group increased. The amount of TNF-α in siRNA-transfected cells was significantly lower than that in the Sc group at each time-point (P<0.01). The qPCR and western blot analysis results suggested that the mRNA and protein levels of NF-κB increased in the lung tissue of ALI after 6 h, then peaked and subsequently decreased; however, levels were still high. In comparison with the sepsis and Sc groups, the expression of NF-κB in the siRNA group decreased, while the levels remained higher than those in the healthy control group. No significant difference in the expression of NF-κB between the sepsis and the Sc groups was identified 12 h after injury. The qPCR and ELISA results suggested that the mRNA and protein levels of TNF-α increased in the lung tissue 6 h after injury, then peaked and subsequently decreased; however, they remained higher than those in the healthy group following 12 h. The expression of TNF-α in the siRNA group decreased in comparison with the sepsis and the Sc groups, and the difference at 6 h was statistically significant (P<0.05). Detection of MMP-9 activity in the lung tissue using gelatin zymography revealed that the protein levels of MMP-9 in the siRNA group decreased in comparison with the sepsis and the Sc groups, and the difference at 6 h was statistically significant (P<0.05). The mRNA and protein levels of MMP-9 in the sepsis group and the Sc were higher than those in the healthy group, and this difference was statistically significant (Fig. 4; P<0.05).

In conclusion, the present study suggested that retrovirus-mediated RNAi technology can be used for gene therapy by targeting NF-κB p65 and inhibiting the activation of NF-κB, thereby inhibiting the release of the inflammatory cytokine TNF-α stimulated by endotoxin. It provides a potential application of RNAi technology for the early prevention and treatment of an excessive inflammatory response in ALI, thus revealing a new path for the control of inflammation following ALI.
